# Essential Oils from Different Plant Parts of *Eucalyptus cinerea* F. Muell. ex Benth. (Myrtaceae) as a Source of 1,8-Cineole and Their Bioactivities

**DOI:** 10.3390/ph4121535

**Published:** 2011-11-25

**Authors:** Sayonara Mendes Silva, Simone Yae Abe, Fábio Seigi Murakami, Gustavo Frensch, Francisco A. Marques, Tomoe Nakashima

**Affiliations:** 1 Programa de Pós-Graduação em Ciências Farmacêuticas, Universidade Federal do Paraná, Campus Jardim Botânico, CEP 80210-170, Curitiba, Paraná, Brazil; E-Mail: tomoenakashima@ufpr.br (T.N.); 2 Graduação em Farmácia, Universidade Federal do Paraná, Campus Jardim Botânico, CEP 80210-170, Curitiba, Paraná, Brazil; E-Mail: simone_abe@yahoo.com.br (S.Y.A.); 3 Departamento de Farmácia, Universidade Federal do Paraná, Campus Jardim Botânico, CEP 80210-170, Curitiba, Paraná, Brazil; E-Mail: fsmurakami@ufpr.br (F.S.M.); 4 Programa de Pós-Graduação em Química, Universidade Federal do Paraná, Centro Politécnico, CEP 81531-990, Curitiba, Paraná, Brazil; E-Mails: gufrensch@gmail.com (G.F.); tic@ufpr.br (F.A.M.)

**Keywords:** *Eucalyptus cinerea*, essential oil, 1,8-cineole, seasonal variation, antimicrobial properties

## Abstract

*Eucalyptus cinerea*, known as silver dollar tree, has few descriptions in traditional medicine. Chemical composition and antimicrobial properties of the essential oils of leaves, flowers and fruits, collected seasonally, were determined by GC/MS and disk diffusion/MIC, respectively. 1,8-Cineole was the main compound, particularly in fresh leaves—Spring (74.98%), dried leaves—Spring (85.32%), flowers—Winter (78.76%) and fruits—Winter (80.97%). Other compounds were found in the aerial parts in all seasons: α-pinene (2.41% to 10.13%), limonene (1.46% to 4.43%), α-terpineol (1.73% to 11.72%), and α-terpinyl acetate (3.04% to 20.44%). The essential oils showed antimicrobial activities against bacteria and yeasts, with the best results being found for the dried autumn and winter leaves oils (MIC < 0.39 mg/mL) against *Streptococcus pyogenes*. For the other tested microorganisms the following MIC results were found: *Staphylococcus aureus*— Dried leaves oil from summer (0.78 mg/mL), *Pseudomonas aeruginosa*—Flowers oil from autumn and fruits oil from winter (1.56 mg/mL) and *Candida albicans*—Flowers oil from autumn and fruits oils from winter and spring (0.78 mg/mL).

## 1. Introduction

*Eucalyptus* belongs to the family Myrtaceae and encompasses approximately 900 species and subspecies [1,2]. The genus is originally from Australia and has spread throughout the World due to its favorable characteristics such as adaptability, ease of cultivation and rapid growth. The main products obtained from the *Eucalyptus* are oil, gum, cellulose and wood, while the essential oil extracted from the leaves is widely employed in the medicine, perfume and food industries [3]; in the former case this is mainly due to its antimicrobial, antifungal, antiseptic, astringent, anti-inflammatory, wound healing, disinfectant, and expectorant properties [4,5].

The incidence of food-borne diseases remains a significant problem, even in the developed World, where approximately 30% of the population of such countries is affected by these diseases every year [6].

Foods containing synthetic additives have been the subject of some suspicion, since it has been suggested that some of these additives can convert ingested materials into toxic or carcinogenic substances by increasing the activity of microsomal enzymes [7]. In this context, the high incidence of health problems related to foods, the pressure from consumers on the food industry to produce foods containing fewer synthetic additives and the search for safer foods have created the driving force to seek new natural food additives.

Extracts of *Eucalyptus* leaves are approved as food additives [8]. The essential oil of eucalyptus is regarded as safe and non-toxic by the United States Food and Drug Authority (FDA). Meanwhile, in Europe the use of eucalyptus essences as aromatizers in foods has also received approval [3]. In Japan, the extract of eucalyptus leaves appears on the List of Food Additives as an antioxidant [9].

A large number of investigations have demonstrated the antimicrobial activity of aromatic oils from the leaves of *Eucalyptus* [2,10,11], although only a few studies have addressed the use of essential oils from other plant organs such as fruits and flowers [12,13]. Studies of its activity against pathogenic microorganisms and those that cause the deterioration of food are also scarce [2].

*Eucalyptus cinerea* F. Muell. ex Benth. is one of the numerous species of the genus and its main use is ornamental, with few reports of it being used in popular medicine [14]. However, among species of Eucalyptus the yield of essential oil from the leaves of *E. cinerea* and that of its main compound 1,8-cineole (eucalyptol) are considered high [14-16]. The high concentrations of essential oil in the leaves, as well as in the flowers and fruits, could potentially be employed for therapeutic ends and as natural additives for use in the food, cosmetics and perfume industries, extending the use of the plant beyond the predominantly ornamental.

Reports describing the antimicrobial properties of *E. cinerea* are scarce; moreover, the antibacterial and antifungal activities of essential oils of its fruits and flowers have not been studied previously. In this context, the objective of the present study was to define the chemical composition of samples of essential oils from the aerial parts—Leaves, flowers and fruits—of *E. cinerea* collected seasonally, as well as to test the antimicrobial activity of its aromatic oils against Gram-positive (*Staphylococcus aureus* and *Streptococcus pyogenes*) and Gram-negative (*Pseudomonas aeruginosa* and *Escherichia coli*) bacteria and yeast (*Candida albicans*).

## 2. Results and Discussion

### 2.1. Extraction and Yield of Essential Oils

The period of extraction for each essential oil of *Eucalyptus cinerea* was six hours, with the first hour returning a better yield, and from the third hour onwards the volume of essential oil extracted was minimal. This finding is in agreement with the data reported for dried leaves oil from *E. cinerea* by Franco *et al*. [17].

The Farmacopéia Brasileira [18] reported the minimum amount of essential oil in leaves of *Eucalyptus globulus* as 0.8% (v/m), with the main compound being 1,8-cineole, the levels of which must exceed 70% in order to be considered a medicinal oil.

**Figure 1:**
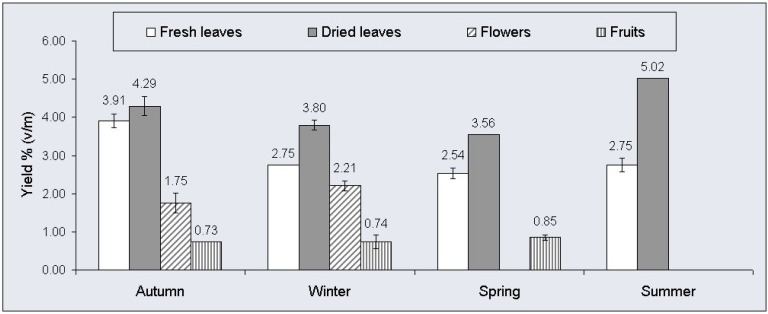
Yield of essential oils from aerial parts of *E. cinerea*.

As shown in Figure 1, the samples of fresh leaves from *E. cinerea* presented lower yields of oils than the corresponding samples of dried leaves, with the essential oil average yield of the summer sample of dried leaves being particularly high at 5.02% (v/m). The amounts of essential oils in both fresh and dried leaves were higher than those reported in the literature for the same species, where yields were 0.26% (v/m) and 2.87% (v/m) [15,16].

The essential oil yields obtained from different dried and fresh plant organs from the plant species studied, over the course of the four seasons of the year, were quite remarkable, in particular for dried leaves in the summer. This fact suggests that further studies with this essential oil are viable, with the possibility of it being employed in industry and other applications.

Essential oil yields are influenced by the season, by the aerial part of the plant collected and by the drying process. In addition, other factors can alter the yield and chemical composition of the essential oils: temperature, water availability, stage of development of the plant, genetic variation, climate, environment, geographical conditions, UV radiation, and soil nutrients, among others [19].

### 2.2. Physico-Chemical Analyses of the Essential Oils

Physico-chemical analyses were carried out to establish parameters for the quality control of the volatile oils of *E. cinerea*. The analyses of the relative density and the refractive index of the essential oils of leaves, flowers and fruits of *E. cinerea* did not reveal any significant variations in relation to the different aerial parts and the seasons (Table 1). Furthermore, the physico-chemical data relating to the dried leaves were in agreement with the findings of Moreira *et al*. [14] and Zrira *et al*. [15].

The results obtained for the solubility in ethanol of the essential oils extracted from fresh and dried leaves, flowers and fruits, over the course of the seasons of the year are presented in Table 2. It can be seen that in all of the samples more than one part volume of 70% ethanol was necessary for the oil to become miscible.

### 2.3. Chemical Composition of the Essential Oils

The results obtained from the analysis of the chemical composition of the essential oils of leaves, flowers and fruits of *E. cinerea* are presented in Table 3. The chemical composition of the essential oils of different parts of the same plant can vary widely [20].

Eucalyptol (1,8-cineole) is the major chemical component of the oils obtained from leaves for the majority of medicinal species of *Eucalyptus*, such as *E. staigeriana* [2], *E. globulus* [11,21], and *E. urophylla* [22]. Nevertheless, the main component in other species of *Eucalyptus* may be a different compound, such as piperitone—*E. dives*; (*E*)-methyl cinnamate—*E. olida* [2], α-pinene—*E. camaldulensis* [22], limonene—*E. staigeriana* [21], β-citronellal—*E. citriodora* [21], and *p*-cymene—*Eucalyptus tereticorni* [23].

The main volatile compound identified in all of the aerial parts of *E. cinerea* collected seasonally was 1,8-cineole, which reached a concentration of 85.32%. In addition to this compound, others that were found included α-pinene, limonene, α-terpineol, and α-terpinyl acetate.

Other studies have shown that the main components of the essential oil of *E. cinerea* obtained from dried leaves from a single period were 1,8-cineole and α-pinene [15]. Franco *et al.* [17] also identified limonene and α-terpineol.

In the essences of fresh leaves of *E. cinerea*, the greatest variation observed for 1,8-cineole was 60.69% to 83.61% in summer and winter, respectively. The other principal compound in this sample of essential oil was α-terpinyl acetate, which varied from 5.38% to 20.44% in the winter and summer, respectively. Meanwhile, in the aromatic oils of dried leaves the highest and lowest amounts of 1,8-cineole and α-terpinyl acetate also were observed in the winter and summer seasons. This finding may be related to the temperature, relative humidity, and the incidence of UV light, as well as other environmental factors [19].

The results obtained by Babu and Singh [16], for the 1,8-cineole content of the species *E. cinerea* (Himalaya region), differed from our data, since they found higher concentrations in fresh leaves than in dried leaves.

The compound δ-3-carene was detected exclusively in flowers both in the autumn and in the winter, and in the same proportions. Meanwhile, 1,8-cineole was the dominant compound, just as in leaves and fruits. According to Giamakis *et al.* [12], who described the chemical composition of the oil of *E. camaldulensis* flowers, the principal components are 1,8-cineole and β-pinene.

In the samples of volatile oils obtained from the fruits of *E. cinerea*, eucalyptol (1,8-cineole) was the predominant component, making it different from the species *E. globulus* in which aromadendrene was the principal compound in the fruits [13].

## 2.4. Antimicrobial Activity of the Essential Oils

### 2.4.1. Disk Diffusion

The disk diffusion test is accepted by the FDA (Food and Drug Administration of the USA) and is established as a standard by the National Committee for Clinical Laboratory Standards (NCCLS) for the analysis of antimicrobial activity in conventional antimicrobial agents such as antibiotics [24]. However, the chemical properties presented by the oils do not permit the standardised methodology to be followed completely. Consequently, modifications were made based on other techniques proposed in the literature [25].

The essential oils of *E. cinerea* besides their pure major compound 1,8-cineole purchased commercially were tested for antimicrobial activity against Gram-positive (*S. aureus, S. pyogenes)* and Gram-negative bacteria (*E. coli*, *P. aeruginosa*), and yeasts (*C. albicans*), as shown in Table 4. The oil from autumn flowers at 100% exhibited greater activity against *P. aeruginosa* (17.0 ± 0.2 mm), whereas the highest degree of inhibition of the bacterium *S. pyogenes* was observed with the essential oil at 100% of fresh leaves collected in summer (26.0 ± 2.1 mm). The halo of inhibition for *S. aureus* (13.0 ± 0.2 mm) was greater than that for the remaining samples when the aromatic oil at 100% from the dried leaves collected in autumn was employed and, by contrast, the highest activity against the yeast was seen with the oil at 100% from the fresh leaves obtained in spring (15.0 ± 0.5 mm). The pure 1,8-cineole presented no antimicrobial activity against *S. aureus* and *C. albicans*.

Through statistical analysis by the Tukey method (P < 0.05) performed with crude essential oils (100%) of *E. cinerea*, significant differences between the averages of the halos of inhibition were observed, as shown in Table 4. Statistical analysis demonstrated that for *S. aureus* and *C. albicans* the inhibitory actions of all the tested samples were significantly higher than the action of the pure compound 1,8-cineole, whereas for *E. coli* only the dried leaves oils from autumn, spring and summer, the flowers oil from autumn and the fruits oils from autumn and spring presented inhibitory actions significantly different, although lower, than that of 1,8-cineole. For *P. aeruginosa* the four crude oils from dried leaves presented inhibitory actions significantly lower than that of 1,8-cineole, whereas all other oils showed actions significantly higher than the action of the pure compound. Regarding the analysis with *S. pyogenes* only the fresh leaves oil from summer, dried leaves oil from winter and flowers oils from autumn and winter showed actions significantly higher than that of 1.8-cineole.

#### 2.4.2. Minimum Inhibitory Concentration—MIC

The disk diffusion method was employed with the objective of obtaining a preliminary assessment of the antimicrobial potential of the pure and diluted essential oils against Gram-positive and Gram-negative bacteria and yeasts. Following this preliminary screening, those essential oil samples whose halos of inhibition exceeded 8 mm were selected for further analysis by the microdilution method [26]. Therefore the microdilution method was not used with the bacterium *E. coli* since this microorganism did not appear to be sensitive to the samples by the disk diffusion method.

The aim of the microdilution method is to determine the MIC of each sample against different microorganisms. This method is widely used due to its sensitivity and the fact that it requires minimal quantities of reagents and samples, which enables a greater number of repetitions and, thus, increases the reliability of the results.

Although the leaves of this plant are the most widely used part due to the fact that they are available throughout the year, the essential oils derived from the flowers and fruits showed themselves to be more effective than the volatile oil of the leaves, presenting MICs of 1.56 mg/mL and 0.78 mg/mL, in autumn and winter, respectively, towards the microorganisms *P. aeruginosa* and *C. albicans* (Table 5). In the tests performed with *S. aureus*, the sample of oil obtained from dried leaves in summer exhibited antimicrobial activity up to a concentration of 0.78 mg/mL, the lowest MIC observed with this strain. Additionally, the samples of aromatic oils from the dried leaves collected in autumn and winter presented a remarkable antimicrobial potential against the strain *S. pyogenes* (MIC < 0.39 mg/mL), with this being the lowest concentration observed in all of the tests carried out.

The analysis for the commercially purchased isolated chemical compound 1,8-cineole presented MIC values against the tested microorganisms much higher than the values of the *E. cinerea* essential oils samples (Table 5).

To our knowledge, this is the first report of an antimicrobial effect for the essential oils of fruits and flowers of *E. cinerea*. The antimicrobial activities of the essential oils varied according to the concentration and the type of bacterium. These differences in the susceptibility of the tested microorganisms to the essential oils may be attributed to a variation in the rate of penetration of the active component of the essential oil through the cell wall and structures of the cell membrane [27].

The Gram-positive bacteria were more susceptible to the essential oils than their Gram-negative counterparts, as a result of their lipopolysaccharide membrane which restricts the diffusion of hydrophobic components [27], consistent with the results observed for samples of *E. cinerea*. The Gram-positive bacteria allow direct contact between the hydrophobic components of the essential oils and the phospholipid bilayer of the cell membrane, where they exert their effects such as an increase in the permeability to ions and the leakage of vital intracellular constituents, or compromise bacterial enzyme systems [28]. Some researchers have reported a relationship between the chemical structures of the most abundant compounds in the essential oils and their antibacterial activity [20].

The marked diversity of chemical groups that comprise essential oils suggests that the antimicrobial activity cannot be attributed to a specific mechanism, but rather to several [29]. On the other hand, these mechanisms do not necessarily represent different targets, with some of them being dependent on others [20]. That behavior can be observed in *E. cinerea* when comparing the results of their essential oils with that of 1,8-cineole alone.

Phenolic compounds are mainly responsible for the antimicrobial action of essential oils; however, there is evidence that minor components of essential oils play a fundamental role in their antimicrobial properties due to synergistic effects [20]. According to the literature compounds such as limonene, linalool, γ-terpinene, *p*-cimene, α-pinene, and α-terpineol also exhibit antimicrobial activity [2,30]. Interestingly, van Vuuren and Viljoen [31] have reported that limonene and 1,8 cineole have synergistic antimicrobial effects.

The principal component responsible for the antimicrobial activity against *S. aureus* was terpineol, which was eight times more active than 1,8-cineole for the species *E. radiata* [11,32]. In some of the samples of essential oil from *E. cinerea* (fresh leaves from summer and fruits from spring) a relatively high concentration of terpineol was found, at 11.72% and 10.80%, respectively.

## 3. Experimental

### 3.1. Plant Material

The plant material—Leaves, flowers and fruits—from the species *E. cinerea* F. Muell. ex Benth., Myrtaceae was collected from some specimens located in the Centro Politécnico—Universidade Federal do Paraná (UFPR), in Curitiba, Paraná, Brazil (latitude 25° 27′ 4.70′′ S, longitude 49° 13′ 52.05′′ W and altitude of 922 m), during the 2009–2010 period. An exsiccate was identified (catalogue number 47.863) and deposited in the herbarium of the Department of Botany (UPCB), Biological Sciences Centre, UFPR.

The collections of the aerial parts from *E. cinerea* were made during the four seasons of the year, in order to investigate the variation in the composition of metabolites between the seasons and also between the different plant organs. Leaves could be collected in the whole year, however it was noted that in spring there was an absence of flowers, while in the summer only leaves were present. For this reason, the plant material from flowers was collected only in autumn and winter, whereas the collections of fruits were performed only in autumn, winter and spring.

The collected leaf material was divided in two samples, one kept at room temperature for fifteen days in order to dry, and the other used fresh immediately after the collection. The whole material from flowers and fruits was used after first being left to dry for fifteen days at room temperature.

### 3.2. Extraction of Essential Oils

The essential oils of *E. cinerea* were extracted from the leaves, flowers and fruits collected in the autumn, winter, spring and summer by hydrodistillation in a Clevenger-type apparatus for a period of six hours [18], using around 200 g of each material fragmented in 2 L of distilled water. Samples of fresh and dried leaves were separated, with the latter being processed fifteen days after collection. The yield of each essential oil was determined as percent volume (mL) of essential oil per mass (g) of plant material (% v/m) [18].

### 3.3. Physico-Chemical Analyses

The following physico-chemical parameters of the essential oils were analysed.

#### 3.3.1. Determination of the Relative Density

The relative density (d_20_^20^) was determined using a 1 cm^3^ capacity pycnometer [18].

#### 3.3.2. Determination of Refractive Index

This assay was carried out in an ABBE ausJENA refractometer, at a temperature of 20 °C [18].

#### 3.3.3. Determination of Solubility in Ethanol

The solubility of the essential oil was determined in ethanol at 70%, 80%, 90%, and in absolute ethanol [18]. This test measures the volume of ethanol required to solubilise 1 volume of essential oil (v/v).

### 3.4. Gas Chromatography Mass Spectrometry (GC/MS)

Gas chromatography combined with mass spectrometry (GC/MS) was employed to identify the volatile constituents present in the essential oil. A Varian^®^ 3800 gas chromatograph was used coupled with a Saturn^®^ 2000 mass spectrometer, equipped with a CP-Sil 8 low bleeding apolar column (30 m × 0.25 mm × 0.25 µm). The carrier gas was helium, used at a constant pressure of 59 kPa and a constant flow of 1 mL/min. The injector temperature was 280 °C, with the initial temperature set at 60 °C and a temperature ramp of 3 °C/min rising to a final temperature of 280 °C for 10 min. The samples of essential oil were diluted at a ratio of 1 µL/mL of hexane.

The identification of compounds in the oils was based on the linear retention index, calculated in relation to the retention times of a homologous series of *n*-alkanes (RI), and on the fragmentation pattern observed in the mass spectra, by comparing these with data in the literature [33] and from the NIST 2008 mass spectral library—System data base. All determinations were performed in duplicate and averaged.

### 3.5. Assessment of Antimicrobial Activity

The following strains of microorganisms were employed: *Staphylococcus aureus* ATCC 6538, *Streptococcus pyogenes* ATCC 19615, *Pseudomonas aeruginosa* ATCC 9027, *Escherichia coli* ATCC 25922 and *Candida albicans* ATCC 10231 from NEWPROV®, all of which were reconstituted according to the supplier's instructions. The microbial cultures were standardised to 10^8^ CFU/mL, estimated by comparison with the 0.5 McFarland standard, and later inoculated in culture media for use in the assessment of antimicrobial activity.

#### 3.5.1. Disk Diffusion Method

The antimicrobial activity of the samples of essential oils from *E. cinerea* and of the pure compound 1,8-cineole (purchased from Sigma-Aldrich) was assessed by the disk diffusion method [25]. In this technique, carried out in a Class II biological safety cabinet, suspensions of *S. aureus*, *P. aeruginosa*, *E. coli*, *S. pyogenes* and *C. albicans* were prepared in 0.9% physiological saline and standardised according to McFarland standards. With the aid of a sterile swab the microbial suspensions were seeded in triplicate, on plates containing Mueller-Hinton agar for bacteria and Sabouraud Dextrose agar for yeasts. Sterile disks of filter paper 6 mm in diameter were impregnated with 10 µL of the samples and placed over the seeded material. The samples of essential oils and of 1,8-cineole were tested as 100%, and also at dilutions of 75%, 50% and 25% in 10% Tween 80. Chloramphenicol (30 µg) and ketoconazole (50 µg) were employed as positive controls, with 10% Tween 80 as a negative control. The plates were transferred to an incubator at 35 °C for 24 h in the case of bacteria and 25 °C for 48 h in the case of *C. albicans*. At the end of the appropriate incubation period for each microorganism, the halos of inhibition around each disk were measured (in mm) and the mean of the results was calculated.

#### 3.5.2. Microdilution Method for Determination of the Minimum Inhibitory Concentration (MIC)

The MIC of the essential oils samples and of the compound 1,8-cineole (Sigma-Aldrich) was determined according to previously published methods with some modifications [26,27]. Those essential oils that presented antimicrobial activity by the disk diffusion method against the microorganisms and produced inhibition halos greater than 8 mm were submitted to the microdilution test in broth, in order to determine the minimum inhibitory concentration (MIC) [26].

For the microorganisms *S. aureus*, *P. aeruginosa*, and *E. coli*, the MIC was determined using Mueller-Hinton broth, while for *S. pyogenes* Tripticase Soy broth was used, and for the yeast *C. albicans* Sabouraud Dextrose broth. The assay was carried out in 96-well flat-bottomed sterile microplates. Each well was first inoculated with a volume of 100 µL of the various samples, prepared at a concentration of 100 mg/mL, diluted in 10% Tween 80. Next, 100 µL of the specific broth for each microorganism were added to each well using a multichannel micropipette. Then, a 100 µL aliquot of the content of each well was transferred to the next well in sequence and, after mixing, the same volume was transferred to the following well, with the procedure being repeated to obtain serial dilutions of 1:2, 1:4, 1:8, 1:16, 1:32, 1:64, 1:128, and 1:256, giving the following decreasing concentrations of samples: 50 mg/mL; 25 mg/mL; 12.5 mg/mL; 6.25 mg/mL; 3.12 mg/mL; 1.56 mg/mL; 0.78 mg/mL; and 0.39 mg/mL. The microbial inocula at a concentration of 0.5 McFarland (10^8^ CFU/mL) were diluted 1:10 in sterile saline solution (0.9%), and from this dilution a volume of 10 µL was added to each well. The diluent, 10% Tween 80, was employed as a negative control, while the antibiotic chloramphenicol, at a concentration of 30 µg/mL, and the antifungal ketoconazole, at 50 µg/mL, were used as positive controls, both being subjected to serial dilution. The microplates were incubated at 35 °C for 24 h, in the case of the bacteria, and at 25 °C for 48 h in the case of *C. albicans*. After this period 20 µL of an aqueous solution of the indicator TTC (triphenyltetrazolium chloride), at 0.5%, were added to each well and the microplates were then incubated for a further hour at 35 °C. The presence of a pink-red colour was interpreted as negative evidence of the inhibitory effect for the microorganism, while a colourless solution was considered positive evidence of the inhibitory action of the sample. The MIC was defined as the lowest concentration of the sample, in mg/mL, able to suppress microbial growth (that is, the appearance of a pink colour). Each test was carried out in triplicate.

## 4. Conclusions

*E. cinerea* is a species that could be employed as a source of 1,8-cineole, since the aerial parts of this plant (leaves, flowers and fruits) revealed themselves to be rich in this compound in all seasons of the year, peaking at a concentration of 85.3% in the essential oil. This study also revealed that the samples of essential oils obtained had antimicrobial potential against Gram-positive and Gram-negative bacteria, and against yeasts, with the most sensitive microorganism being *S. pyogenes*, followed by *S. aureus*, *P. Aeruginosa* and *C. albicans*, while *E. coli* was the most resistant, in the preliminary screening using the disk diffusion method. Determination of the minimum inhibitory concentration (MIC) of the essential oils revealed better results for the oils of the dried leaves collected in autumn and in winter, both of which exhibited values below 0.39 mg/mL against *S. pyogenes*. This study demonstrates, for the first time, the antimicrobial effect of the essential oils of fruits and flowers of *E. cinerea*. Considering the antibacterial properties and antifungal activity of these essential oils, they show promise for applications in foods, pharmaceutical products and cosmetics.

## Figures and Tables

**Table 1:** Relative density and refractive index of essential oils from leaves, flowers and fruits of *Eucalyptus cinerea*.

	**Fresh Leaves**				**Dried Leaves**				**Flowers**		**Fruits**		
	**AUT**^a^	**WIN**^b^	**SPR**^c^	**SUM**^d^	**AUT**	**WIN**	**SPR**	**SUM**	**AUT**	**WIN**	**AUT**	**WIN**	**SPR**
Relative density	0.912	0.899	0.907	0.904	0.913	0.900	0.909	0.908	0.910	0.899	0.909	0.908	0.901
Refractive index	1.459	1.458	1.459	1.460	1.460	1.459	1.458	1.460	1.460	1.460	1.464	1.463	1.460

a Autumn.

b Winter.

c Spring.

d Summer.

**Table 2:** Solubility in ethanol of essential oils from plant organs of *E. cinerea*.

**Ethanol Concentration**	**Fresh Leaves**				**Dried Leaves**				**Flowers**		**Fruits**		
	**AUT**^a^	**WIN**^b^	**SPR**^c^	**SUM**^d^	**AUT**	**WIN**	**SPR**	**SUM**	**AUT**	**WIN**	**AUT**	**WIN**	**SPR**
70%	1:3	1:3	1:3	1:5	1:3	1:3	1:2	1:5	1:3	1:3	1:3	1:3	1:4
80%	1:1	1:1	1:1	1:1	1:1	1:1	1:1	1:1	1:1	1:1	1:1	1:1	1:1
90%	1:1	1:1	1:1	1:1	1:1	1:1	1:1	1:1	1:1	1:1	1:1	1:1	1:1
100%	1:1	1:1	1:1	1:1	1:1	1:1	1:1	1:1	1:1	1:1	1:1	1:1	1:1

a Autumn.

b Winter.

c Spring.

d Summer.

**Table 3:** Chemical composition (%) of essential oils from aerial parts of *E. cinerea*.

**Compound**	**RI**^a^	**Fresh leaves**				**Dried leaves**				**Flowers**		**Fruits**		
		**AUT**^c^	**WIN**^d^	**SPR**^e^	**SUM**^f^	**AUT**	**WIN**	**SPR**	**SUM**	**AUT**	**WIN**	**AUT**	**WIN**	**SPR**
α-pinene	940	3.55	4.97	3.44	2.41	4.02	5.73	3.13	3.10	4.83	8.24	10.03	10.13	6.19
δ-3-carene	1014	-	-	-	-	-	-	-	-	1.11	1.10	-	-	-
*o*-cymene	1033	-	0.39	-	0.07	0.47	-	-	0.40	2.31	1.45	3.61	1.98	2.32
limonene	1038	3.20	3.32	2.07	2.35	4.43	3.29	1.46	3.78	3.72	2.98	3.29	2.16	1.86
1,8-cineole	1041	74.59	83.60	74.98	60.69	73.93	83.95	85.32	69.96	73.07	78.76	71.58	80.97	62.58
NI^b^	1186	-	-	-	-	-	-	-	-	-	-	1.00	-	-
NI	1187	-	-	-	-	-	-	-	-	-	-	-	-	0.70
γ-terpineol	1193	0.14	-	-	0.91	-	-	-	0.38	1.50	-	0.79	-	1.77
NI	1199	-	-	-	-	-	-	-	-	-	-	-	-	0.57
α-terpineol	1207	5.88	1.73	6.11	11.72	4.88	1.28	2.59	6.98	4.70	1.78	4.42	1.72	10.80
methyl geranate	1325	-	-	-	0.98	0.17	-	-	0.11	-	-	-	-	-
α-terpinyl acetate	1360	12.64	5.38	12.57	20.44	11.11	4.82	7.23	14.50	8.76	5.69	5.28	3.04	13.21
prezizaene	1443	-	0.61	0.83	0.43	0.99	0.93	0.27	0.79	-	-	-	-	-

a Retention index relative to *n*-alkane series on the CP-Sil 8 low bleeding apolar column;

b Not identified;

c Autumn;

d Winter;

e Spring;

f Summer.

**Table 4:** Measure of inhibition halos (mm) obtained by disk diffusion method (average of three replicates ± standard deviation).

**Microorganisms**	**Conc.**^a^	**Fresh leaves**			**Dried leaves**				**Flowers**		**Fruits**			**1,8-cineole**
		**Autumn**	**Spring**	**Summer**	**Autumn**	**Winter**	**Spring**	**Summer**	**Autumn**	**Winter**	**Autumn**	**Winter**	**Spring**	
*S. aureus* ATCC 6538	100%	11.5 ± 0.5	11.0 ± 0.1	10.5 ± 0.5	13.0 ± 0.2	12.5 ± 1.5	10.0 ± 0.1	12.0 ± 0.1	11.0 ± 0.1	7.0 ± 0.1	7.0 ± 0.1	10.5 ± 0.5	9.5 ± 0.5	0.0
		A	A	A,B	A	A	A,B	A	A	A	B	A,B	A,B	C
	75%	9.0 ± 0.1	9.0 ± 0.1	8.5 ± 0.5	11.0 ± 0.1	11.0 ± 0.2	9.5 ± 0.5	16.0 ± 0.0	9.5 ± 0.5	11.0 ± 1.0	9.0 ± 0.2	10.0 ± 0.0	10.0 ± 1.0	0.0
	50%	7.5 ± 0.5	7.0 ± 0.1	7.0 ± 0.1	9.5 ± 0.5	8.5 ± 0.5	8.0 ± 0.1	15.0 ± 0.0	9.0 ± 0.1	10.5 ± 0.5	7.0 ± 0.0	8.0 ± 0.1	9.0 ± 1.0	0.0
	25%	0.0	0.0	0.0	6.8 ± 0.3	6.5 ± 0.1	6.5 ± 0.1	8.0 ± 0.0	7.0 ± 0.0	7.5 ± 0.5	0.0	7.0 ± 0.0	7.3 ± 0.8	0.0
	PC1^b^	27.5 ± 0.6	28.0 ± 0.3	27.5 ± 0.5	30.5 ± 0.5	28.0 ± 0.3	28.5 ± 0.5	28.5 ± 0.5	31.0 ± 0.2	31.0 ± 0.1	28.5 ± 0.5	28.0 ± 0.2	28.5 ± 0.5	28.5 ± 0.5
	NC^d^	0.0	0.0	0.0	0.0	0.0	0.0	0.0	0.0	0.0	0.0	0.0	0.0	0.0
*S. pyogenes* ATCC 19615	100%	10.5 ± 1.5	9.5 ± 0.5	26.0 ± 2.1	11.0 ± 1.0	23.5 ± 2.5	12.0 ± 0.2	14.0 ± 0.2	21.0 ± 1.0	20.5 ± 4.5	14.0 ± 0.1	16.0 ± 1.0	16.5 ± 0.5	9.0 ± 0.2
		A	A	B	A	B,C	A,D	A,D	B,C,D	B,C,D	A,D	A,C,D	A,C,D	A
	75%	8.0 ± 0.1	8.0 ± 0.1	12.0 ± 0.2	9.0 ± 0.1	14.0 ± 1.0	9.0 ± 0.2	9.5 ± 0.5	13.5 ± 0.5	18.0 ± 2.0	12.5 ± 0.5	12.5 ± 0.5	9.0 ± 0.2	0.0
	50%	7.0 ± 0.1	7.0 ± 0.1	10.5 ± 0.5	7.5 ± 0.5	10.0 ± 1.0	8.0 ± 0.1	8.0 ± 0.1	12.0 ± 2.0	14.0 ± 0.0	10.0 ± 2.0	8.0 ± 0.1	7.0 ± 0.1	0.0
	25%	6.5 ± 0.0	0.0	9.0 ± 0.1	0.0	0.0	0.0	7.5 ± 0.5	10.0 ± 0.0	12.0 ± 2.0	0.0	6.8 ± 0.3	0.0	0.0
	PC1	46.5 ± 1.5	46.5 ± 1.5	37.0 ± 1.0	46.5 ± 1.5	35.5 ± 0.5	46.5 ± 1.5	46.5 ± 1.5	40.0 ± 4.0	52.5 ± 2.5	33.0 ± 0.2	46.5 ± 1.5	46.5 ± 1.5	35.0 ± 0.1
	NC	0.0	0.0	0.0	0.0	0.0	0.0	0.0	0.0	0.0	0.0	0.0	0.0	0.0
*P. aeruginosa* ATCC 9027	100%	14.5±0.6	14.0±0.1	14.0±0.2	0.0	0.0	0.0	0.0	17.0±0.2	16.0±0.2	12.0±0.1	14.0±0.1	13.5±0.5	8.5±0.5
		A	A	A	B	B	B	B	C	C	D	A	A	E
	75%	12.0 ± 1.0	11.5 ± 0.5	11.0 ± 0.1	0.0	0.0	0.0	0.0	15.5 ± 0.6	14.5 ± 0.5	10.0 ± 0.1	12.5 ± 0.6	10.5 ± 0.5	7.0 ± 0.1
	50%	9.0 ± 0.1	9.5 ± 0.5	9.0 ± 0.1	0.0	0.0	0.0	0.0	14.0 ± 2.0	13.0 ± 0.1	8.0 ± 0.0	9.0 ± 1.0	9.5 ± 0.5	0.0
	25%	7.0 ± 0.0	6.8 ± 0.3	7.0 ± 0.0	0.0	0.0	0.0	0.0	9.5 ± 0.5	10.0 ± 0.0	6.5 ± 0.0	7.5 ± 0.5	0.0	0.0
	PC1	28.5 ± 0.6	26.0 ± 0.3	27.5 ± 0.5	21.0 ± 0.2	17.0 ± 1.0	22.5 ± 0.5	20.0 ± 2.0	32.5 ± 1.0	32.0 ± 2.0	28.5 ± 0.5	29.0 ± 0.2	28.0 ± 1.0	29.5 ± 0.5
	NC	0.0	0.0	0.0	0.0	0.0	0.0	0.0	0.0	0.0	0.0	0.0	0.0	0.0
*E. coli* ATCC 25922	100%	8.0 ± 0.1	8.0 ± 0.1	8.0 ± 0.1	7.0 ± 0.1	8.0 ± 0.1	7.0 ± 0.1	7.0 ± 0.0	7.0 ± 0.1	8.5 ± 0.5	0.0	8.0 ± 0.1	7.0 ± 0.0	8.0 ± 0.2
		A	A	A	B	A	B	B	B	A	C	A	B	A
	75%	7.0 ± 0.1	7.3 ± 0.3	7.0 ± 0.1	6.5 ± 0.1	7.0 ± 0.1	6.5 ± 0.1	6.5 ± 0.0	0.0	0.0	0.0	0.0	0.0	0.0
	50%	0.0	0.0	0.0	0.0	6.5 ± 0.1	0.0	6.5 ± 0.0	0.0	0.0	0.0	0.0	0.0	0.0
	25%	0.0	0.0	0.0	0.0	0.0	0.0	0.0	0.0	0.0	0.0	0.0	0.0	0.0
	PC1	31.0 ± 1.0	31.0 ± 0.2	31.0 ± 1.0	32.5 ± 0.5	28.5 ± 0.5	32.5 ± 1.5	26.0 ± 0.0	33.5 ± 1.5	33.0 ± 0.2	30.5 ± 0.5	30.5 ± 0.5	33.5 ± 1.5	28.5 ± 0.5
	NC	0.0	0.0	0.0	0.0	0.0	0.0	0.0	0.0	0.0	0.0	0.0	0.0	0.0
*C. albicans* ATCC 10231	100%	14.0 ± 1.0	15.0 ± 0.5	14.0 ± 0.1	12.0 ± 0.1	11.5 ± 0.5	11.5 ± 0.5	12.5 ± 0.5	14.0 ± 2.0	11.0 ± 0.2	9.0 ± 0.1	10.0 ± 1.0	9.5 ± 0.6	0.0
		A,B	A	A,B	A,B,C	A,B,C	A,B,C	A,B,C	A,B	A,B,C	C	B,C	C	D
	75%	12.0 ± 0.1	11.0 ± 1.0	11.0 ± 0.2	8.5 ± 0.5	9.5 ± 0.5	10.0 ± 1.0	11.0 ± 0.2	9.5 ± 0.5	7.0 ± 0.1	7.0 ± 0.1	8.0 ± 0.1	8.0 ± 1.0	0.0
	50%	9.0 ± 0.0	10.0 ± 0.1	0.0	7.0 ± 0.0	8.0 ± 0.1	8.5 ± 0.6	8.5 ± 0.5	7.0 ± 0.1	7.0 ± 0.0	6.5 ± 0.1	7.0 ± 0.1	7.3 ± 0.8	0.0
	25%	0.0	0.0	0.0	0.0	0.0	0.0	0.0	0.0	0.0	0.0	0.0	0.0	0.0
	PC2^c^	23.0 ± 1.0	22.0 ± 0.2	22.5 ± 0.5	24.0 ± 0.1	24.5 ± 0.5	24.0 ± 1.1	26.0 ± 1.0	25.5 ± 0.5	24.0 ± 1.0	24.0 ± 0.2	25.0 ± 0.1	26.0 ± 0.1	25.0 ± 0.2
	NC	0.0	0.0	0.0	0.0	0.0	0.0	0.0	0.0	0.0	0.0	0.0	0.0	0.0

a Concentration.

b Positive control for bacteria (chloramphenicol 30µg/disk).

c Positive control for yeast (ketoconazole 50 µg/disk).

d Negative control (10% Tween 80). For 100% concentration, averages followed by same capital letter (A,B,C,D,E) in the row do not differ significantly by Tukey test (P < 0.05).

**Table 5:** MIC (mg/mL) of essential oils from leaves, flowers and fruits of *E. cinerea* and of 1,8-cineole.

**Microorganism**	**Fresh Leaves**			**Dried Leaves**				**Flowers**		**Fruits**			**1,8-cineole**
	**Autumn**	**Spring**	**Summer**	**Autumn**	**Winter**	**Spring**	**Summer**	**Autumn**	**Winter**	**Autumn**	**Winter**	**Spring**	
*S. aureus*	3.12	3.12	12.50	3.12	3.12	1.56	0.78	3.12	3.12	6.25	3.12	3.12	50.00
*S. pyogenes*	3.12	1.56	6.25	<0.39	<0.39	3.12	1.56	3.12	1.56	1.56	1.56	1.56	50.00
*P. aeruginosa*	3.12	3.12	3.12	*	*	*	*	1.56	3.12	3.12	1.56	3.12	>50.00
*E. coli*	*	*	*	*	*	*	*	*	*	*	*	*	25.00
*C. albicans*	1.56	1.56	6.25	3.12	3.12	3.12	1.56	0.78	1.56	1.56	0.78	0.78	12.50

* MIC not performed because the inhibition halos < 8 mm in disk diffusion method; Average obtained from three replicates with standard deviation zero.
